# Subunits Med12 and Med13 of Mediator Cooperate with Subunits SAYP and Bap170 of SWI/SNF in Active Transcription in *Drosophila*

**DOI:** 10.3390/ijms252312781

**Published:** 2024-11-28

**Authors:** Yulii V. Shidlovskii, Yulia A. Ulianova, Alexander V. Shaposhnikov, Valeria V. Kolesnik, Anna E. Pravednikova, Nikita G. Stepanov, Darya Chetverina, Giuseppe Saccone, Lyubov A. Lebedeva, Victor K. Chmykhalo, Ennio Giordano

**Affiliations:** 1 Laboratory of Gene Expression Regulation in Development, Institute of Gene Biology, Russian Academy of Sciences, 119334 Moscow, Russiavkchmykhalo@icloud.com (V.K.C.); 2 Department of Biology and General Genetics, Sechenov University, 119992 Moscow, Russia; 3 Group of Epigenetics, Institute of Gene Biology, Russian Academy of Sciences, 34/5 Vavilov St., 119334 Moscow, Russia; daria.chetverina@gmail.com; 4 Department of Biology, University of Naples “Federico II”, 80126 Naples, Italy

**Keywords:** SAYP, Bap170, Med12, Med13, transcription, promoter, enhancer, gene expression

## Abstract

SAYP and Bap170, subunits of the SWI/SNF remodeling complex, have the ability to support enhancer-dependent transcription when artificially recruited to the promoter on a transgene. We found that the phenomenon critically depends on two subunits of the Mediator kinase module, Med12 and Med13 but does not require the two other subunits of the module (Cdk8 and CycC) or other subunits of the core part of the complex. A cooperation of the above proteins in active transcription was also observed at endogenous loci, but the contribution of the subunits to the activity of a particular gene differed in different loci. The factors SAYP/Bap170 and Med12/Med13 did not form sufficiently stable interactions in the extract, and their cooperation was apparently local at regulatory elements, the presence of SAYP and Bap170 in a locus being necessary for stable recruitment of Med12 and Med13 to the locus. In addition to the above factors, the Nelf-A protein was found to participate in the process. The cooperation of the factors, independent of enzymatic activities of the complexes they are part of, appears to be a novel mechanism that maintains promoter activity and may be used in many loci of the genome. Extended intrinsically disordered regions of the factors were assumed to sustain the mechanism.

## 1. Introduction

The control of gene expression is performed primarily at the transcription initiation stage. In higher eukaryotes, several dozens of different coactivator complexes have been described to change the state of the chromatin template at regulatory elements and to form a pre-initiator complex at the promoter [1]. Chromatin remodeling complexes, which change the position of nucleosomes and ensure efficient recruitment of DNA-binding factors, are the most important regulators of the chromatin state [2]. SWI/SNF (Switch/sucrose non-fermentable), or Brahma, is one of the most abundant families and is represented by two major subfamilies, BAF (BRG1/BRM-associated factor) and PBAF (Polybromo-associated BAF). Analysis of the SWI/SNF distribution on the genome has shown that the BAF family occurs mainly on active enhancers, whereas PBAF is localized on promoters in human cells [3]. Subunits specific to the families seem to determine the specificity of their recruitment to different sites in the genome. In *Drosophila*, the homologous PBAP (Polybromo-associated Brahma-associated Protein complex) subfamily includes the PB (Polybromo)/Bap180, SAYP (Supporter of Activation of *Yellow* Protein), and Bap170 (Brahma-associated protein 170 kD) subunits. SAYP and Bap170 are partners and interact with various transcriptional activators, such as DHR3 (*Drosophila* Hormone Receptor 3) and STAT (Signal Transducer and Activator of Transcription) [4,5]. In the mammalian PBAF complex, all three homologs of these proteins, PBMR1 (Polybromo-1), PHF10 (PHD Finger Protein 10), and ARID2 (AT-rich Interactive Domain 2), form contacts with the core of the complex [6].

Mediator is another key coactivator involved in the formation of the pre-initiator complex. The complex ensures the contact between enhancers and promoters and acts as a coordinating hub around which almost all participants in the transcription process assemble. Mediator interacts with both general transcription factors and chromatin-modifying complexes and numerous gene-specific activators. Mediator is a large protein complex with more than 20 subunits. The subunits combine into four main structural modules. A dissociable kinase module stands out among them and consists of four subunits: Cdk8 (Cyclin-dependent kinase 8), CycC (Cyclin C), Med12, and Med13. The module attaches to the central core of Mediator via its Med13 and Med12 subunits [7]. The module is involved in interactions with transcription factors and can apparently be recruited to a gene independently.

Coactivator activities provide precise control of gene activity in the cell. However, the coordination of different groups of coactivators and the dynamics of their recruitment to chromatin have not yet been sufficiently studied [8,9]. We have previously discovered a new mechanism whereby the SAYP and Bap170 subunits participate in enhancer-dependent transcription (Figure S1) [10,11]. A specific model system used in the study is based on a driver transgene expressing a LexA DBD (DNA-binding domain) fusion with either SAYP or Bap170. Another component of the system is the *LacZ* reporter under the control of the minimal *hsp70* promoter supplemented with *lexAop* binding sites. The reporter was inserted at multiple loci around the fly genome; some of the insertion sites were close to active enhancers. We found that both a nearby active enhancer and recruitment of the lexA fusion are necessary for the activation of the *lacZ* reporter. Thus, being anchored via the LexA DBD–LexAop system to the promoter of the *LacZ* reporter, SAYP and Bap170 render the promoter capable of capturing the signal from a nearby enhancer and forming a contact with the enhancer. Knockdown experiments have shown that the phenomenon is independent of other SWI/SNF subunits in the case of Bap170. We used the model of *LacZ* activation by the *Dad* gene enhancer in imaginal discs to further investigate the underlying molecular mechanisms of reporter activity.

## 2. Results

### 2.1. Screening of Factors Regulating SAYP, Bap170-Dependent Transcription

Using the model established previously [11], we performed a search for other transcription factors that potentially play a role in *LacZ* reporter activation. To this end, we have used the fly lines that express hairpin RNAs complementary to mRNAs of various transcription factors under the control of the UAS sequence. In the present study, we mainly focused on the subunits of the Mediator complex, which is pivotal in the enhancer–promoter interaction. We studied additionally the promoter-associated factors Transcription Factor IIB (TFIIB) and several subunits of the TFIID complex, which is associated with SAYP [12]. Finally, we analyzed the RNA polymerase II pause factors DRB Sensitivity-Inducing Factor (DSIF) and Negative Elongation Factor (NELF), which are also associated with SAYP [13], and the SWI/SNF-associated factor GAGA Factor (GAF) [14].

Visualization of the knockdown effect in our model is easily performed by imaginal discs’ staining, as we have shown previously [11]. Thus, the *en-GAL4* driver active in the posterior part of the imaginal disc was used. Screening results are summarized in Supplementary Table S1.

Knockdowns of the factors Med6, Med7, Med14, Med17, and TBP-associated factors TAF1 and TAF5 resulted in the failure of larval imaginal discs to develop normally. It can be concluded that knockdowns of these factors were effective and decreased the levels of the respective proteins to significantly impair development. However, it was impossible to assess their contribution to the activity of the *LacZ* transgene.

The use of hairpins complementary to mRNAs of the Med10, Med18, Med25, and Med31 subunits had no phenotypic manifestation and exerted no effect on the expression of the *LacZ* transgene under the control of SAYP. The hairpins might act inefficiently or might not be expressed, and the interpretation of this group of results is therefore ambiguous.

Knockdowns of the factors Med1, Med15, Med20, Med21, Med27, Med28, Cdk8, CycC, TFIIB, DSIF, Nelf-E, and Nelf-D did not affect *LacZ* transgene activity under the control of the lexA–SAYP fusion. However, lethality at the larval, pre-pupal, or pupal stage was observed for these lines (Supplementary Table S1), indicating the efficiency of the hairpins (in the case of the Med15, DSIF, and Cdk8 hairpins, a knockdown under the control of *en-GAL4* led to wing defects, but lethality was observed when the *tub-GAL4* ubiquitous driver was used). The above subunits belong to different modules of the Mediator complex. Thus, contrary to expectations, most of the Mediator subunits were not essential for the effect of the enhancer on the transgene in our system.

Finally, we identified a group of hairpins whose expression reduced *LacZ* reporter activity and was lethal, like the knockdowns described above. The group included Med12, Med13, Med16, and Nelf-A. Knockdowns of the above three Mediator subunits disrupted *LacZ* transgene expression both under lexA–SAYP control and under lexA–Bap170 control. Interestingly, a knockdown of the GAF factor increased transgene expression under the control of LexA–SAYP and caused wing defects in adult insects (Supplementary Table S1). A similar effect has previously been observed for a Mor subunit knockdown [11]. It is possible that the GAF knockdown alters chromatin accessibility and promotes more efficient recruitment of the fusion protein to chromatin.

Because the four subunits of the Mediator kinase module showed opposite effects on *LacZ* transgene activity, we examined their effects in more detail.

### 2.2. Med12 and Med13 Subunits, but Not Cdk8 and CycC, Are Required for SAYP/Bap170-Dependent Transgene Activity

**Figure 1:**
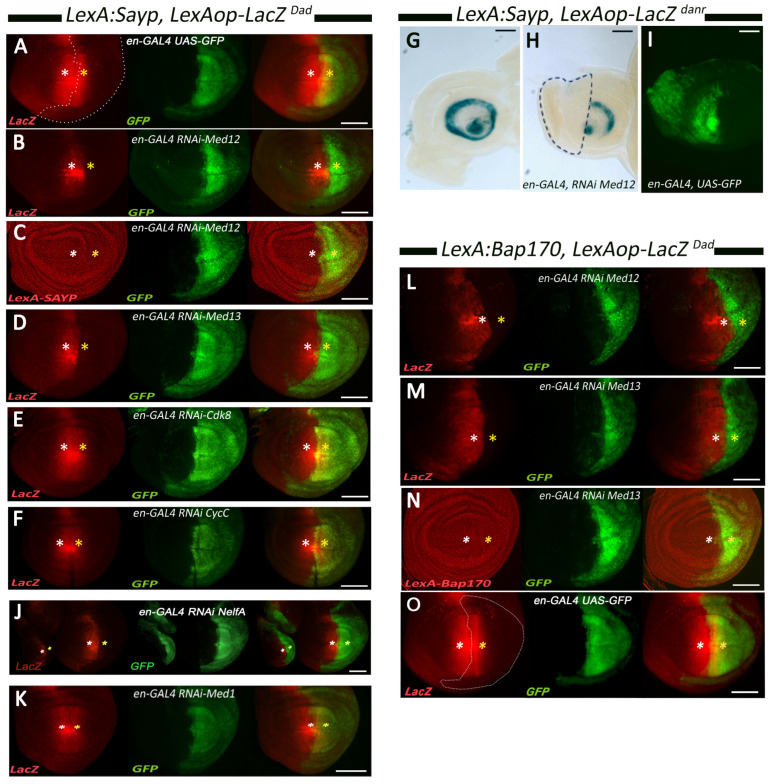
SAYP- and Bap170-mediated enhancer-dependent transgene expression requires the mediator kinase module subunits Med12 and Med13 but not CycC or Cdk8. (**A**–**K**) Fluorescence imaging of the SAYP-mediated trapping of the *Dad* enhancer in wing discs from larvae with the genotype *en-GAL4*, *UAS-GFP*, *LexA-SAYP*, *LexAop-LacZ^Dad^*. The *LexA-SAYP*, *LexAop-LacZ^Dad^* transgenes were expressed alone (control in (**A**)) or in combination with RNAi transgenes for Med12 (**B**,**C**), Med13 (**D**), Cdk8 (**E**), CycC (**F**), Nelf-A (**J**), and Med1 (**K**). Lack of Med12 or Med13 efficiently downregulates the reporter (**B**,**D**), respectively, whereas downregulation of Med12 does not affect the level of the LexA–SAYP fusion protein (**C**). Downregulation of Cdk8 or CycC in the posterior region of the wing discs does not affect *LexAop-LacZ^Dad^* transgene expression (as shown by the yellow overlapping region in (**E**,**F**)). A knockdown of Nelf-A shows a phenotype similar to that in a Med12/Med13 knockdown (**J**), while a Med1 knockdown showed no effect (**K**), like in the case of Cdk8/CycC depletion. (**G**–**I**) β-Gal activity in antennal discs from larvae with the genotype *en-GAL4*; *LexA-SAYP, LexAop-LacZ^danr^* were expressed alone (**G**) or in combination with RNAi for Med12 (**H**). The SAYP-mediated trapping of the *danr* (*distal antenna-related*) enhancer on the *LexAop-LacZ^danr^* transgene is abolished in a cell clone lacking Med12 (a region within a dotted line in (**H**)). (**I**) An expression pattern of *en-GAL4* in an antennal disc marked by UAS-GFP. (**L**–**O**) Wing discs from larvae with the genotype *en-GAL4*, *UAS-GFP*; the *LexA-Bap170*, *LexAop-LacZ^Dad^* in combination with RNAi for Med12 (**L**) or Med13 (**M**,**N**); a control is shown in (**O**). LexA–Bap170-mediated enhancer-dependent activation is abolished by the downregulation of Med12 (**L**) or Med13 (**M**). LexA–Bap170 accumulation is not affected by lack of Med13 (**N**) in the posterior region of the wing disc. White asterisks indicate the anterior regions with normal *LexAop-LacZ^Dad^* transgene expression (outside the RNAi induction region), and yellow asterisks indicate the posterior regions overlapping the regions of RNAi activation. Scale bar, 100 μm.

As previously shown, the LexA–SAYP/Bap170 fusion proteins are capable of activating *LexAop-hsp70* promoter–*LacZ* reporter expression following the pattern of the nearby enhancer of the *Dad* gene in our model [11]. Expression of *LacZ* is detected as a band of cells in both posterior and anterior parts of the imaginal wing disc (Figure 1A).

A knockdown of the Med12 subunit in the posterior part of the imaginal disc silenced the transgene; i.e., *LacZ* expression was undetectable in the region of *en-GAL4* driver expression (Figure 1B). This was not a result of a low level of *LexA–SAYP* transgene expression given that LexA–SAYP fusion protein levels were not affected in the entire disc (Figure 1C). Similarly, a knockdown of Med13 significantly reduced the level of transgene expression (Figure 1D). Knockdowns of the other two subunits of the kinase module, Cdk8 and CycC, had no effect on transgene expression (Figure 1E,F). A knockdown of Nelf-A (Supplementary Table S1) caused a phenotype similar to that of the Med12 knockdown, whereas a knockdown of Med1 had no effect, as was the case with Cdk8 (Figure 1J,K, respectively).

We checked if the described effect is *Dad* enhancer specific or reflects a general feature of SAYP/BAP170 interaction with Med12/Med13 in transcriptional regulation. We tested the effect of the Med12 knockdown on another transgene obtained earlier; its activation was controlled by another enhancer located at a different position in the genome [11]. In that case, *LacZ* expression was under the control of LexA–SAYP and the *Danr* gene enhancer and was detected by a specific pattern in antennal discs (Figure 1G). The *en-GAL4* driver was used for a knockdown. Knockdown of Med12 led to a specific decrease in transgene activity in the region of driver activation (Figure 1H,I). Thus, the Med12 knockdown effect is independent of the enhancer nature.

The Med12 and Med13 knockdowns were additionally tested for effect on the second fusion protein in LexA–Bap170-dependent expression of *LacZ* under the control of the *Dad* enhancer in imaginal discs. As in the case of LexA–SAYP-dependent expression, the knockdown of Med12 or Med13 blocked transgene activity (Figure 1L,M). At the same time, the expression level of the *LexA–Bap170* transgene was not affected (Figure 1N).

This result is quite unexpected, as we have previously shown that a knockdown of other SWI/SNF subunits does not affect LexA–Bap170-dependent *LacZ* activity in this system [11]. A knockdown of subunits of the other complex has a more significant effect than a knockdown of the immediate neighborhood of Bap170, the result emphasizing the crucial role of the Med12 and Med13 subunits in Bap170-dependent transgene activation.

Control experiments were performed to test the knockdown efficiency. We used the *Ms1096-GAL4* wing disc driver with a well-defined expression pattern to visualize the efficiency of the Med12 knockdown. Staining showed efficient depletion of Med12 according to the driver expression pattern (Figure S2A), indicating that the knockdown was efficient. Expression of the fusion protein LexA–Bap170 remained intact upon Med12 depletion in this system (Figure S2B), like with the *en-GAL4* driver. Examination of the Med13 expression pattern in wing discs after a Med13 knockdown with *en-GAL4* in the posterior region confirmed the knockdown efficiency; i.e., much weaker staining with antibodies to Med13 was observed in the posterior region (Figure S2C). Moreover, the fly stocks expressing hairpins against the Med12, Med13, and Nelf-A mRNAs were used in other studies [15,16,17]; the fact further underlines the specificity of the knockdowns used. To study the knockdown efficiency of the Cdk8 and CycC subunits, we used their known target gene *E(spl)mβ* because appropriate antibodies were unavailable [18]. Both Cdk8 and CycC knockdowns using the *en-GAL4* driver decreased the expression of the target gene in the particular region of driver activity (Figure S2D–F).

Thus, the knockdowns of the four subunits of the kinase module were efficient and did not affect the expression of the LexA–SAYP and LexA–Bap170 fusion proteins.

**Figure 2:**
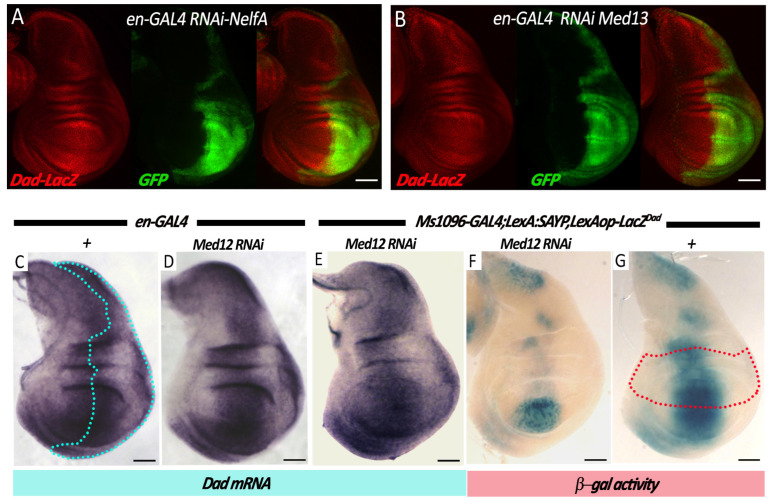
Med12/Med13 are specifically required to act at the tethered *LexAop-LacZ ^Dad^* transgene but are dispensable for *Dad* enhancer/promoter activity. (Top panel) A possible dependence of the *Dad* enhancer regulation by Nelf-A and Med13 was monitored in wing discs of larvae with the genotype *en-GAL4*, *Dad-LacZ*, using a *Dad-LacZ* enhancer trap line in combination with an RNAi line for Nelf-A or Med13 ((**A**) and (**B**), respectively). In both cases, the communication of the *Dad* enhancer with the P-element promoter of the *Dad-LacZ* insertion was never compromised as observed by comparing the β-gal accumulation between the anterior and posterior regions of the wing pouch. (Bottom panel) The *Dad* promoter efficiency was monitored by analyzing the *Dad* mRNA levels by in situ hybridizations in wing discs of larvae with the genotype *en-GAL4* alone as a control (**C**) or in combination with RNAi for Med12 (**D**). (**E**–**G**) Wing discs of larvae with the genotype *Ms1096-GAL4; LexA–SAYP* and *LexAop-LacZ^Dad^* alone (**G**) or in combination with the Med12 RNAi insertion (**E**,**F**) were used to analyze the *Dad* mRNA level (**E**) and the activity of the *LexAop-LacZ* transgene (**F**,**G**). Scale bar, 100 μm.

We tested additionally whether the activity of the *Dad* gene enhancer itself is maintained in larvae after the knockdowns (Figure 2). For this purpose, the activity of the *Dad* enhancer was evaluated in an enhancer-trap system in a fly line that carried a P-element insertion near the *Dad* gene and showed a *LacZ* expression pattern similar to the expression pattern of the *Dad* gene (the transgene is hereafter referred to as *Dad-LacZ*) [19]. We have previously tested the effects of knockdowns of SWI/SNF subunits in this model and shown that reducing the level of SAYP, Bap170, Mor, or Brm does not alter the pattern of *Dad*-enhancer activity in imaginal discs [11] (Supplementary Table S1).

*Dad*-enhancer activity was tested upon a knockdown of the Med13 subunit using the *en-GAL4* driver. Expression of the *Dad-LacZ* transgene remained uniform throughout the disc, indicating that enhancer activity and *Dad* gene expression were insensitive to the knockdown (Figure 2B). A Nelf-A knockdown also did not disrupt *Dad*’s activity (Figure 2A). The effect of a Med12 subunit knockdown on *Dad* gene expression was also tested directly. In situ hybridization showed a normal expression pattern when Med12 levels were reduced with the *en-GAL4* driver (Figure 2D). A Med12 knockdown was additionally performed using the *Ms1096*-*GAL4* driver (Figure 2F,G). Again, the knockdown did not disrupt the *Dad* gene expression (Figure 2E). However, the expression of the *LexAop-LacZ* transgene under the control of LexA–SAYP (Figure 2G) decreased according to the expression pattern of the driver (Figure 2F).

Similarly, we tested the activity of the *Dad* enhancer upon knockdowns of the Med1, Med4, Med15, Med27, Med28, and DSIF subunits and showed that the activity remained intact (Supplementary Table S1). The finding is consistent with the data on the effect of knockdowns on *LacZ* expression (see Section 2.1). This confirms the specificity of the results obtained.

Thus, the knockdowns of the Med12 and Med13 subunits were specific, did not impair the very ability of the *Dad* enhancer to activate transcription, and did not affect the expression levels of the LexA–SAYP and LexA–Bap170 fusions. The effect of the Med12 and Med13 knockdowns consists of specific disruption of the communication between active *Dad* and *danr* enhancers and *LexAop*-*hsp70* promoter mediated by LexA–SAYP or LexA–Bap170. The knockdowns of the Cdk8 and CycC subunits were efficient but did not show such an effect.

### 2.3. SAYP/Bap170 and Med12/Med13 Subunits Have Similar Localization in the Genome and Have Similar Functional Effects on Genes

**Figure 3:**
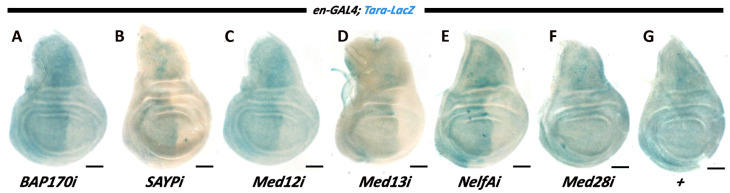
Expression of *taranis* in wing discs upon RNAi-mediated depletion of the PBAP and Med12/Med13 subunits. Wing discs from larvae with the genotype *en-GAL4*, *tara-LacZ* alone or in combination with the RNAi transgene indicated. Ubiquitous expression of *taranis* in the larval wing disc is affected in the posterior region upon RNAi activation in the case of RNAi for Bap170 (**A**), SAYP (**B**), Med12 (**C**), Med13 (**D**), and Nelf-A (**E**), but not for the proper Mediator complex subunit Med28 (**F**); a control is shown in (**G**). Scale bar, 100 μm.

Artificial recruitment of SWI/SNF to the transgene was used in the experiments described above. The cooperation of its SAYP/Bap170 subunits and the Med12 and Med13 subunits of the Mediator kinase module was additionally studied without using LexA-mediated recruitment to the loci. We chose the *tara* gene, which belongs to the trithorax group and is presumably under the control of SWI/SNF [20]. To study the expression profile of *tara*, we used another enhancer-trap line, in which *LacZ* was under the control of *tara* enhancers (hereafter referred to as *tara-LacZ*) (Figure 3) [21]. *LacZ* expression in imaginal discs was examined in wild-type larvae and larvae knocked down for various factors. In the wild type, *LacZ* showed ubiquitous expression in discs (Figure 3G).

A knockdown of SAYP or Bap170 using the *en-GAL4* driver suppressed *tara-LacZ* expression in the posterior region (Figure 3A,B). It is noteworthy that a knockdown of Med12 or Med13 showed the same effect and suppressed gene expression (Figure 3C,D). A knockdown of the Nelf-A factor decreased *tara* activity, like in the case of the LexAop transgenes (Figure 3E). At the same time, a knockdown of the Med28 subunit, which did not affect the expression of the *LexAop-LacZ* transgene as described above, did not affect the expression of *tara-LacZ* (Figure 3F). Knockdowns of the factors Med1, Med4, Med27, Cdk8, DSIF, and NELF-D also had no effect on *tara-LacZ* expression (Supplementary Table S1). Thus, the above subunits behaved similarly in the *tara-LacZ* and *LexAop-LacZ^Dad^* transgene models. The *tara* enhancers acted in a SWI/SNF-dependent manner, in contrast to the *Dad* enhancer. However, in both cases, Med12 and Med13, but not the other Mediator subunits, were found to be critical for gene activity under the control of these enhancers.

Testing the knockdowns of the Med16 and GAF subunits showed that they did not affect *tara-LacZ* gene activity, although their knockdowns affected *LexAop-LacZ* transgene expression. Thus, only three factors—Med12, Med13, and Nelf-A—showed similar behavior in the two models described.

**Figure 4:**
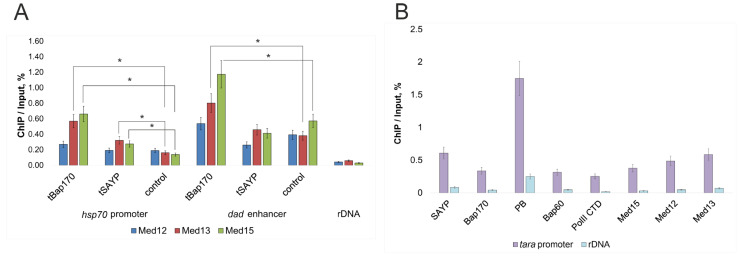
ChIP analysis of the recruitment of various factors to regulatory elements in the models used. (**A**) Recruitment of Med12, Med13, and Med15 onto the *hsp70* reporter promoter of *LexAop-LacZ^Dad^* transgene and at the *Dad* enhancer in flies expressing LexA–Bap170 (designated as tBap170), LexA–SAYP (designated as tSAYP), or no LexA (control) were taken for the analysis. (**B**) Recruitment of the indicated proteins onto the *tara* promoter in wild-type flies. PolII, RNA polymerase II. rDNA was used as a control. * *p* < 0.05.

To test whether the observed effects of depletion of the SAYP, Bap170, Med12, and Med13 subunits on gene expression are direct, we studied the recruitment of the subunits to gene regulatory elements by using chromatin immunoprecipitation (ChIP). First, we checked the *hsp70* promoter and *Dad* enhancer in *LexAop-LacZ^Dad^* flies. We have previously shown that SWI/SNF subunits are recruited to the active *LexAop-hsp70* reporter promoter and the *Dad* enhancer (although the activity of the latter is not reduced by knockdowns of SWI/SNF subunits) [11]. Using antibodies against Mediator subunits, the subunits were detected on the transgene *hsp70* promoter and the *Dad* enhancer (Figure 4A) in *LexAop-LacZ^Dad^* flies. We detected both the Med15 subunit, whose knockdown did not affect transgene expression, and the Med12 and Med13 subunits, which were critical for transgene activity. Thus, despite the presence of all Mediator subunits in the system studied, not all of them are essential for transgene activity.

The study of the *tara* gene promoter also showed the recruitment of SWI/SNF and Mediator subunits and the presence of RNA PolII, as was expected for an active gene (Figure 4B). Thus, the factors SAYP, Bap170, Med12, and Med13 are physically present in the regulatory elements of the genes and are most likely directly involved in the control of their activity in the two models.

**Figure 5:**
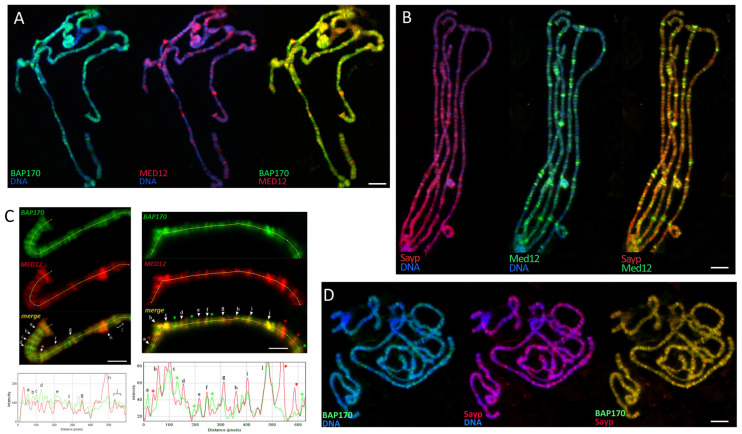
Distribution of Bap170 and Med12 (**A**), SAYP and Med12 (**B**), or SAYP and Bap170 (**D**) on *Drosophila* salivary gland polytene chromosomes visualized by indirect immunofluorescence with antibodies against Bap170, SAYP, and Med12. DNA was stained with DAPI (blue). Split and merged images for red and green channels are shown. (**C**) Details of the Bap170 and Med12 distributions in two different polytenic regions, which were scanned for signal intensity using ImageJ 1.48 software in either red (Med12) or green (Bap170) channels. In graphs, below each polytenic region, examples of bands that contain only Med12 (red asterisks), only Bap170 (green asterisks), or both (black letters) are indicated above the graph. The lines used for the intensity scanning are indicated. Scale bar, 10 (**A**,**B**,**D**) or 5 (**C**) μm.

Potential genome-wide colocalization of SAYP and Bap170 with Med12 and Med13 was studied by immunostaining of salivary gland polytene chromosomes in third-instar larvae. The factors were quite often detected in the same loci of open chromatin, although their distribution profiles differed and there were few loci predominantly enriched in only one of the factors (Figure 5). The finding indicates that the cooperation of SAYP and Bap170 with Med12 and Med13 can potentially be genome-wide.

### 2.4. SAYP/Bap170 Subunits Are Required for the Recruitment of Med12/Med13 to Active Loci in the Genome

To test the mutual influence of the SAYP/Bap170 and Med12/Med13 subunits upon their binding to chromatin, we used the ecdysone-dependent gene *E75*, which has well-characterized enhancers (EcREs) [22,23] and carries subunits of SWI/SNF and the Mediator kinase module on regulatory elements [24]. We induced the gene with ecdysone in S2 cell culture and tested the recruitment of the subunits to the most active promoter and enhancer of the *E75* gene. All of the subunits were detected on the elements. Knockdowns of the subunits under study were obtained to clarify their mutual influence. A knockdown of Med12 did not affect the recruitment of the SAYP/Bap170 subunits to both the *E75* enhancer and promoter. As a control, we checked the recruitment of the core Mediator subunit Med15. Its level was insensitive to the Med12 knockdown as well.

**Figure 6:**
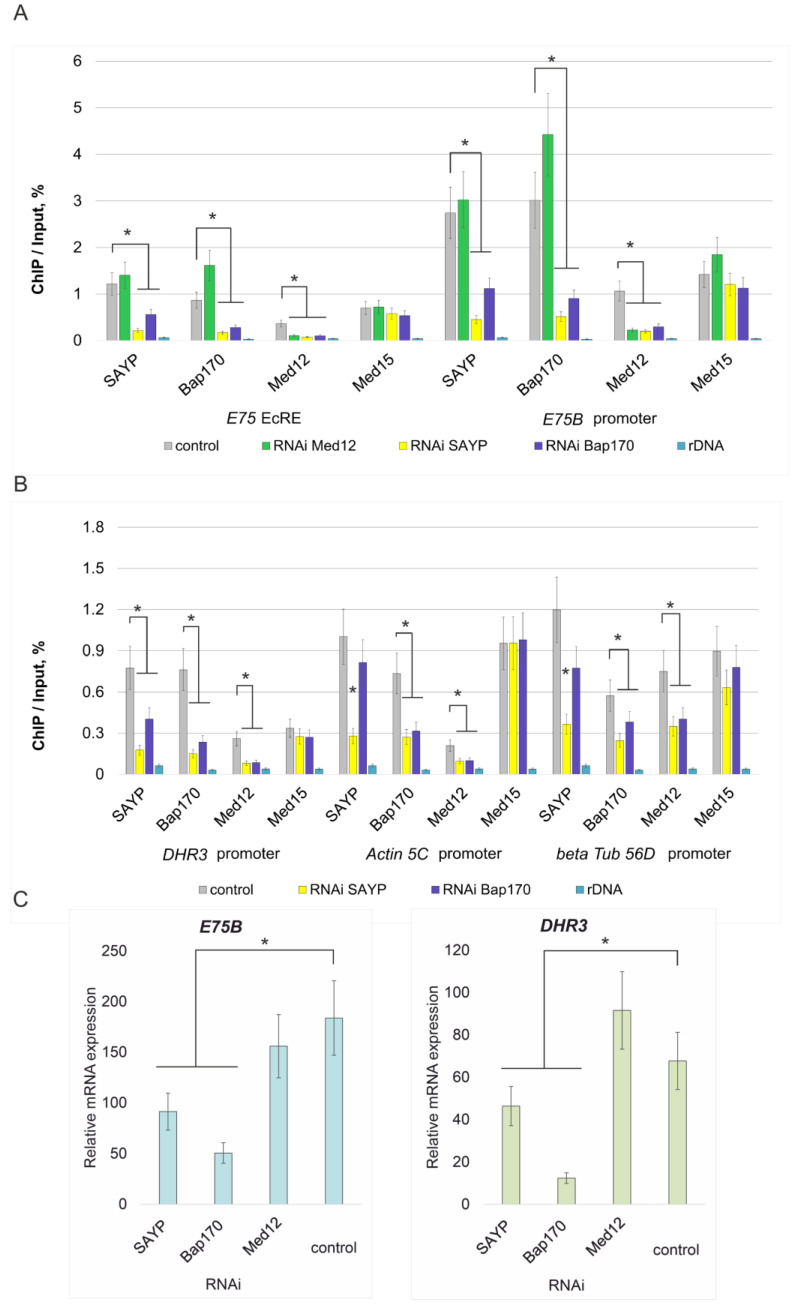
Effects of SAYP, Bap170, and Med12 knockdowns on factor recruitment and gene induction. (**A**,**B**) ChIP analysis of the recruitment of the indicated factors to regulatory elements of the *E75* gene (**A**) or the *DHR3*, *actin 5C*, and *beta-tubulin 56D* gene promoters (**B**) in S2 cells. Knockdowns of the indicated factors were performed. For ChIP analysis of *E75* or *DHR3*, S2 cells were treated with ecdysone. (**C**) Induction of *E75B* and *DHR3* expression by ecdysone in cells knocked down in the indicated factors. The activity of the gene prior to induction was taken as 1. In a control sample, cells were treated with nonspecific GFP dsRNA. * *p* < 0.05.

In an opposite experiment, knockdowns of SAYP and Bap170 strongly affected each other and each significantly impaired the recruitment of Med12. The level of Med15 at the elements was insensitive to the SAYP/Bap170 knockdowns (Figure 6A).

To further check the dependence of Med12 recruitment on SAYP/Bap170, we extended our analysis to include the other ecdysone-dependent gene *DHR3*. Using the same knockdowns, we showed that the presence of the SAYP/Bap170 subunits on the *DHR3* promoter is crucial for Med12, but not Med15, recruitment (Figure 6B). Finally, we checked the promoters of the *actin 5C* and *beta-tubulin 56D* housekeeping genes and detected the same effect; i.e., Med12 recruitment crucially depended on the presence of SAYP/Bap170 (Figure 6B). Thus, this effect takes place at both inducible and basal regulatory elements in the genome.

Induction of *E75* and *DHR3* gene expression was slightly affected by a knockdown of Med12, while strongly depending on Bap170 (Figure 6C). This distinguishes these genes from the transgene models described above. Apparently, a more complex set of molecular mechanisms is responsible for transcription activation of the *E75* gene, in contrast to a simpler activation mechanism of the *LacZ* transgenes.

**Figure 7:**
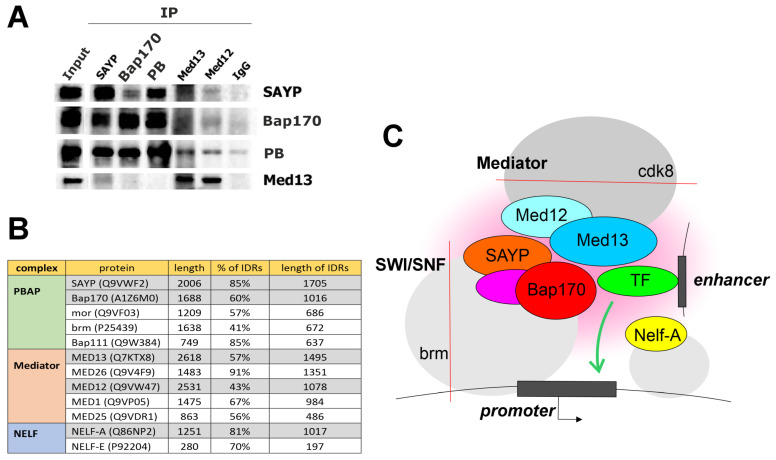
Interaction of the SAYP/Bap170 and Med12/Med13 subunits. (**A**) Immunoprecipitation from an embryonic nuclear extract was performed with the antibodies indicated at the top; Western blot analysis, with the antibodies indicated on the right. (**B**) Top subunits with the longest IDRs in the PBAP, Mediator, and NELF complexes. The total length in amino acid residues, the percentage of IDRs, and the total length of IDRs in amino acid residues are indicated. IDRs were predicted by AlphaFold DB [https://alphafold.ebi.ac.uk/]. (**C**) The hypothesis of a new mechanism whereby the SAYP/Bap170 and Med12/Med13 factors participate in active transcription. SAYP and Bap170 localized on gene regulatory elements contribute to the recruitment of the Mediator subunits Med12 and Med13. Together with TFs localized on enhancers, they presumably form local interactions and a microenvironment required for the active promoter function. Enzymatic activities of the remodeler and Mediator (Brm ATPase and Cdk8 kinase, respectively) are not essential for this mechanism. Presumably, the Nelf-A factor is also a component of this network and is required for efficient enhancer-driven transcription by this mechanism.

To investigate whether co-recruitment of Med12/Med13 and SAYP/Bap170 is due to their physical interaction, immunoprecipitation was performed using an embryonic nuclear extract. Antibodies against SAYP, Bap170, Med12, and Med13 efficiently precipitated the corresponding proteins and direct partners within the same complex (SAYP–Bap170 and Med12–Med13). However, the co-precipitation of subunits of different complexes was hardly detectable (Figure 7A and Figure S5). Thus, the interaction, if any, is rather weak or temporary.

## 3. Discussion

Our previous [11] and current work has revealed a new aspect of the involvement of subunits of coactivator complexes in transcription activation. We have previously found that the recruitment of the SAYP and Bap170 remodeler subunits to the minimal *hsp70* promoter makes it susceptible to the action of a nearby enhancer. In the present work, we found that this phenomenon critically depends on the presence of another component, the Med12 and Med13 subunits of the Mediator kinase module. The presence of other Mediator subunits is not essential in this model system. Using another model of transgenes expressed under the control of the P-element promoter without artificial recruitment of SAYP and Bap170, we similarly found that the presence of SAYP, Bap170, Med12, and Med13 but not other Mediator subunits is essential for enhancer-dependent promoter activity. Thus, the novel mechanism described here consists of tight cooperation of several subunits of functionally divergent complexes in supporting gene activation.

However, when endogenous loci were examined, we did not see such a striking dependence of gene expression on the presence of the subunits. It is possible that the mechanism based on the cooperation of the SAYP/Bap170 and Med12/Med13 factors to maintain active transcription is one of a number of other mechanisms that work at active promoters, but its relative contribution to gene activity is gene-specific.

The question as to how prevalent the mechanism under study is in the genome remains open. Both coactivators are widely distributed throughout the genome, and the mechanism can be expected to contribute to regulating the activities of a large number of genes. It is known that the Mediator is a central factor of signal transduction in many signaling pathways, and the subunits of the kinase module play the most important role in this process [25,26,27,28,29]. A similar function has been assumed for the SWI/SNF complex [30]. Our ChIP experiments showed that the recruitment of the Med12/Med13 subunits depends on the presence of SAYP/Bap170 not only on inducible genes of the ecdysone cascade but also on constitutive housekeeping genes. It is conceivable that co-occurrence of SAYP/Bap170 and Med12/Med13 may be observed at many loci.

Interestingly, artificial recruitment of the SAYP and Bap170 subunits to the *hsp70* transgene promoter does not significantly change the chromatin structure at the promoter according to ChIP H3 and FAIRE data [11]. This indicates that the effect is independent of the enzymatic activity of the remodeler. A knockdown of Brm has no effect on the activity of the promoter when Bap170 is anchored to it, the finding also confirming the assumption. On the kinase module side, we see the involvement of only the structural subunits Med12 and Med13 and the independence of promoter activation from the catalytic subunit of the Cdk8 module. These facts indicate that the SAYP/Bap170 and Med12/Med13 proteins participate in the described phenomenon most likely in a structural rather than catalytic manner. It is noteworthy that ATPase-independent functions of SWI/SNF in *Drosophila* were found to be widespread [31].

Structural and functional relationships of the two Mediator and SWI/SNF complexes have already been observed in various models from yeast to human cells [32,33,34,35,36,37,38]. However, the cooperation of the SAYP/Bap170 and Med12/Med13 subunits, independent of the enzymatic activities of the complexes, was shown for the first time.

It should also be noted that the existence of specific functions that are played by the Med12 and Med13 subunits and differ from those of Cdk8 and CycC has been described widely. In mammalian cells, Cdk8 and Med12 sometimes cooperate on separate genes but act independently in other situations [39,40,41,42]. Kinase-independent cooperation of Med12 with the p300-Jmjd6/Carm1 coactivator has been described and is important for the activation of super-enhancers [43]. Genetic data in *Drosophila* also point to functional differences between the Med12–Med13 and CycC–Cdk8 subunit pairs [44,45,46]. Gene expression analysis has shown that knockdowns of the pairs often have opposite effects on gene expression [47]. The question as to whether Med12 and Med13 protein fractions exist outside the kinase module requires additional study. Recently, the Med13 [48] and CycC [49] proteins have been described to have functions in the cytoplasm that they appear to perform independently of the kinase module. However, large-sized proteins can exhibit polyfunctionality when acting as part of a complex and participate in a number of functions.

The proteins SAYP, Bap170, Med12, and Med13 are multidomain proteins of about 200 kDa, and the presence of extended unstructured domains is their common feature. The domains have been described as part of the Med12 and Med13 subunits [7,50], ARID family proteins (to which the Bap170/ARID2 factor belongs) [51,52,53,54], and other subunits of the SWI/SNF complex [55,56,57]. Extended N-terminal low-complexity domains are a distinctive feature of the SAYP factor, but not its vertebrate homolog PHF10 [58]. Analysis of the distribution of intrinsically disordered regions (IDRs) among the subunits of the PBAP and Mediator complexes showed that the factors under study possess some of the longest IDRs (Figure 7B). The contribution of the IDRs and the related phenomenon of local phase transition to the regulation of transcription is being actively studied. In the case of SWI/SNF, the IDR domains have been shown to contribute to the formation of local condensates with other factors, the formation of a network of protein–protein interactions, localization of factors in the genome, and the fine-tuning of remodeling activity.

We hypothesize that the SAYP/Bap170 and Med12/Med13 factor pairs jointly stimulate a local phase transition and the formation of a microenvironment on a promoter to make the promoter active and susceptible to enhancer action. On the enhancer side, the microenvironment may involve associated transcription factors (Figure 7C). Phase-transition interactions have some specificity, which, however, is significantly lower than the specificity of classic protein–protein interactions. This may explain the lack of association with Med1, which also carries extended IDRs. In addition, this probably explains the fact that Bap170 has a certain level of specificity when anchored to the *hsp70* promoter, i.e., although the promoter can interact with a wide range of enhancers, a number of enhancers do not activate it. For example, the promoter selects the distant *Dpp* disc enhancer, but not the proximal *oaf* gene regulatory sequences, and is incapable of interacting with regulatory elements of the *glec* gene, which is expressed at the dorsal/ventral boundary of the wing discs [59], or the *hsromega* gene, which is widely expressed in larval tissues.

The proteins that produce phase condensates form short-lived local interactions. Their coprecipitation from extracts is therefore inefficient, as was observed in our experiments as well. More sensitive methods should be employed to detect their interactions.

We found additionally that the Nelf-A subunit contributes significantly to the activities of the model transgenes. In *Drosophila*, NELF generally supports enhancer-dependent transcription [60,61]. The mechanism whereby enhancer RNA interacts with Nelf-A and stimulates polymerase pause removal has been described [62,63]. Nelf-A is also a multidomain protein and carries long IDRs [64] (Figure 7B). It is possible that this factor also plays a structural role in the organization of transcription at active promoters.

Coactivators have been the focus of modern molecular genetics for many years. However, details of their co-activation require further study. Our artificial system of transgene activation allowed us to detect a new network of subunits of different complexes contributing to active transcription. Further studies are necessary to perform in order to identify other contributors and the mechanism of their action.

## 4. Materials and Methods

### 4.1. Drosophila Strains

The following GAL4 and UAS lines were used: en-GAL4, UAS-GFP; Ms1096-GAL4; UAS-GFP; UAS-RNAi-SAYP [Vdrc105946]; UAS-RNAi-BAP170 [Vdrc34582]; UAS-RNAi-MED1 [VDRC13054]; UAS-RNAi-MED4 [VDRC101362]; UAS-RNAi-MED6 [BL33743]; UAS-RNAi-MED7 [BL34663]; UAS-RNAi-MED10 [BL34031]; UAS-RNAi-MED12 [VDRC23143, BL34588], UAS-RNAi-MED13 [BL34630]; UAS-RNAi-MED14 [BL34575]; UAS-RNAi-MED15 [VDRC21809]; UAS-RNAi-MED16 [BL34012]; UAS-RNAi-MED17 [VDRC105264]; UAS-RNAi-MED18 [VDRC105264]; UAS-RNAi-MED20 [BL34577]; UAS-RNAi-MED21 [VDRC109982]; UAS-RNAi-MED25 [VDRC108249]; UAS-RNAi-MED27 [VDRC106703]; UAS-RNAi-MED28 [VDRC108282]; UAS-RNAi-MED31 [BL34574]; UAS-RNAi-cdk8 [VDRC107187]; UAS-RNAi-cycC [BL33753, VDRC48835]; UAS-RNAi-TAF1 [BL32421]; UAS-RNAi-TAF4 [VDRC109640]; UAS-RNAi-TAF5 [VDRC45957, VDRC45955]; UAS-RNAi-TBP [VDRC109756]; UAS-RNAi-TFIIB [VDRC106688]; UAS-RNAi-DSIF [VDRC108459]; UAS-RNAi-nelfA [VDRC106245]; UAS-RNAi-Th1 (NELF-D) [VDRC100009]; UAS-RNAi-Nelf-E [VDRC21009]; UAS-RNAi-GAF [VDRC106433]. The RNAi lines were chosen by their ability to induce clear defects with tubulin-GAL4 and en-GAL4 drivers, as well as with other GAL4 lines according to the literature (see Supplementary Table S2). The P{lacW}Dad^j1E4^ was used as a Dad-LacZ enhancer trap [19]. The P{PZ}tara^03881^ line was used as the tara-LacZ enhancer trap line [21]. The P{m8-LacZ}1 line was used as an E(spl)mb enhancer trap line [65]. The transgenic lines expressing LexA–Bap170 (tBap170) and LexA–SAYP (tSAYP) were described previously [11].

### 4.2. Antibodies

Primary antibodies against beta-gal (Promega, Madison, WI, USA), PolII CTD (Abcam, Cambridge, UK), SAYP [58], Bap170 [66], PB [67], and Bap60 [68] were used. In immunostaining experiments, antibodies against Med12 and Med13 (kindly gifted by Jessica Treisman) were used at a 1:500 dilution. In ChIP and IP experiments, polyclonal antibodies against Med12 (fragment 1743–1846 of isoform A), Med13 (fragment 753–881 of isoform C), and Med15 (fragment 1–133 of isoform A) were used (Supplementary Figure S3). Immunofluorescence experiments were performed with rat Alexa Fluor 568 anti-mouse IgG and Alexa Fluor 488 anti-rabbit IgG secondary antibodies (Thermo Fisher, Waltham, MA, USA).

### 4.3. β-Gal Staining, In Situ Hybridization, and Immunofluorescence

Detection of β-gal activity for LacZ reporters was carried out according to standard protocols. Images were captured using either a Leica MZ stereomicroscope or a Reichert-Jung Polyvar microscope using incident fiber optic lights. In situ hybridizations were carried out as described in [69], with a DIG-RNA probe prepared using pBluescript-cloned PCR-amplified fragments corresponding to the white exon. Indirect immunofluorescences were carried out according to standard protocols. In situ hybridizations were carried out as described in [69]. At least 20 larvae were taken for each staining.

### 4.4. Polytene Chromosome Analysis

*Drosophila* polytene chromosome spreads were prepared from salivary glands of the third-instar larvae grown at 17 °C. Spread preparation, fixation, denaturation, hybridization, and immunofluorescence analysis of polytene chromosomes were performed according to a published protocol [70].

### 4.5. S2 Cell Culture, Ecdysone Treatment, and RNAi

*Drosophila* S2 cells were grown in Schneider’s medium (Sigma, Darmstadt, Germany) supplemented with 10% fetal bovine serum (HyClone, Wilmington, DE, USA) and 1× penicillin/streptomycin (HyClone) at 25 °C. Once a week, cells were passaged at a 1:10 dilution and maintained by simple pipetting. For the experiment, cells were collected on the 4th day of cultivation.

S2 cells were counted and plated at 5 × 10^6^ cells in 2 mL of the medium per well in each plate, treated with 1 µM 20E overnight or left untreated [71,72]. RNAi experiments followed published procedures [73]. Treatment with dsRNA (20 μg per million cells) continued for 5 days. In a control experiment, dsRNA corresponding to a GFP coding sequence was used. Primers for dsRNA were listed in the Supplement. The efficiencies of the SAYP, Bap170, Med12, and Med15 RNAi knockdowns are presented in Supplement Figure S4.

### 4.6. Chromatin Immunoprecipitation

ChIP assays were performed as described in [74,75]. S2 cells were cross-linked with 1% formaldehyde (Sigma). Cross-linking was quenched with 125 mM glycine (final concentration). The cells were centrifuged and resuspended in a sonication buffer (50 mM HEPES-KOH [pH 7.9], 140 mM NaCl, 1 mM EDTA, 1% Triton X-100, 0.1% sodium deoxycholate, 0.1% SDS) supplemented with a protease inhibitor cocktail (Roche, Rotkreuz, Switzerland). Chromatin was fragmented by sonication using a Bandelin Sonopuls 3100 HD ultrasonic homogenizer with 20 s bursts repeated 20 times. Chromatin immunoprecipitation was performed using anti-SAYP, anti-Bap170, anti-PB, anti-Med12, and anti-Med15 antibodies, as well as IgG controls. Approximately 2 μg of each antibody was used. The input and immunoprecipitated fractions were analyzed by RT-PCR with appropriate primers. Primers for qPCR are provided in the Supplement and were described previously [74,75].

### 4.7. Western Blotting and Immunoprecipitation

Nuclear extracts from 0 to 12 h *Drosophila* embryos were prepared as described [76]. Embryos were homogenized in an NU-1 solution (15 mM HEPES-KOH [pH 7.6], 10 mM NaCl, 5 mM MgCl_2_, 0.1 mM EDTA, 0.5 mM EGTA, 0.35 M sucrose, 1 mM DTT, 0.2 mM PMSF, 1x PIC) using an overhead stirrer and a Teflon Dounce homogenizer. The suspension was centrifuged at 10,000× *g* for 15 min. Nuclei were resuspended in a NU-2 buffer (15 mM HEPES-KOH [pH 7.6], 110 mM NaCl, 5 mM MgCl_2_, 0.2 mM EDTA, 1 mM DTT, 0.2 mM PMSF, 1x PIC) and extracted with 0.4 M ammonium sulfate. Chromatin was removed by centrifugation at 35,000× *g* for 1 h. Antibodies used in WB were diluted 1:500, and each WB experiment was repeated at least twice. IP was performed as described [70]. DNase I (1 U/μL) and RNase A (10 μg/μL) (Thermo Fisher Scientific, Waltham, MA, USA) were added to an IP buffer at a 1:1000 dilution. Image acquisition and quantification were performed using a ChemiDoc imaging system and ImageLab 1.48 software (Bio-Rad, Hercules, CA, USA).

## Data Availability

Data are contained within the article and Supplementary Materials.

## Supplementary Materials

The following supporting information can be downloaded at https://www.mdpi.com/article/10.3390/ijms252312781/s1. The supplement contains references to publications that utilized various dsRNA-expressing fly stocks used in this work [15,16,17,77,78,79,80,81,82,83,84,85].
