# Supplementary material for: Subunits Med12 and Med13 of Mediator Cooperate with Subunits SAYP and Bap170 of SWI/SNF in Active Transcription in Drosophila
Source: Int J Mol Sci. 2024 Nov 28;25(23):12781. doi: 10.3390/ijms252312781 (PMC11641163; doi:10.3390/ijms252312781)
Supplement: Supplementary file 1 [file ijms-25-12781-s001.zip › ijms-3309786-supplementary.pdf]

Subunits Med12 and Med13 of Mediator Cooperate with Subunits SAYP and Bap170 of SWI/SNF in Active Transcription in Drosophila

Shidlovskii YV at al.

Supplementary Figures

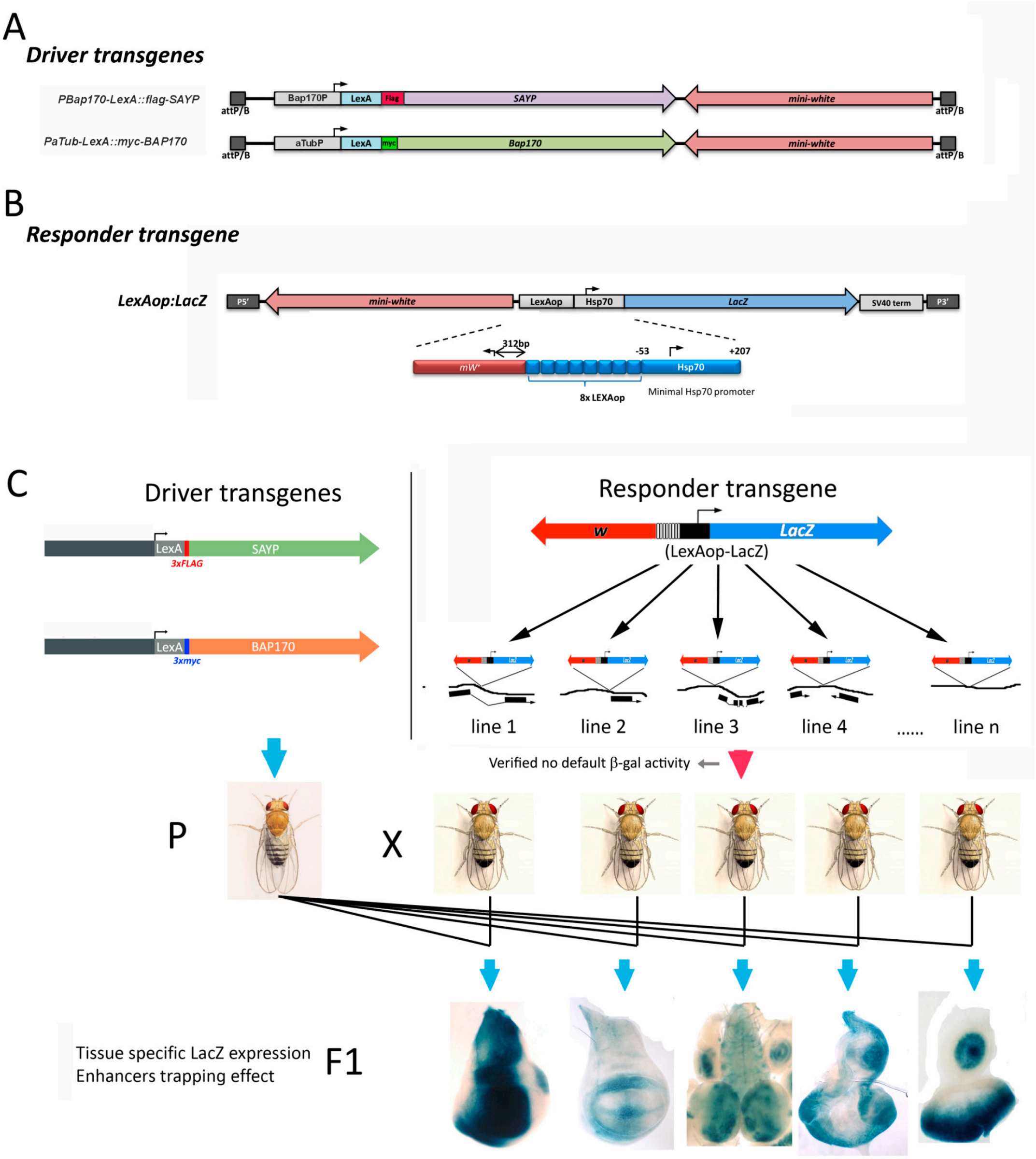

Figure S1. Scheme of the model system to check Bap170 or SAYP for participation in enhancer-dependent transcription [11].
(A) Driver transgenes expressing the LexA-BAP170 or LexA-SAYP fusion proteins. The promoter of the *BAP170* gene or the *alpha-tubulin 84B* gene was used for expression.
(B) The P element-based LexAop:LacZ reporter transgene.The core *hsp70* promoter (−44 to +204) fused with 8x upstream E. coli LexA repressor binding elements was used in the transgene.
(C) Flies carrying insertion of the transgene (B) in different genomic loci were crossed with the driver lines carrting the transgene (A). The progeny shows tissue-specific *lacZ* expression depending of the enhancer located close to the *lacZ* transgene.

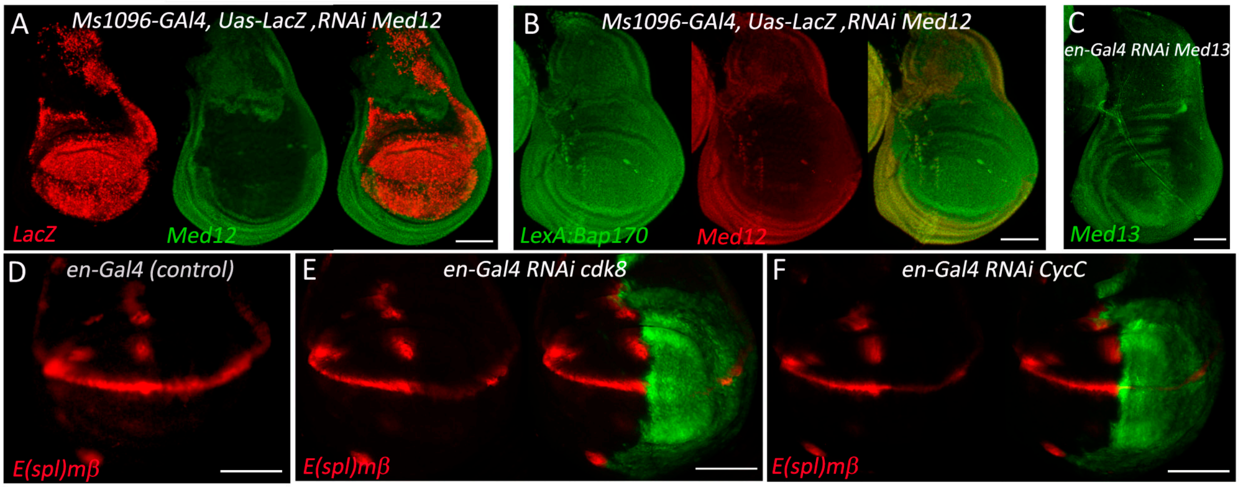

Figure S2. Efficiencies of the *Med12*, *Med13*, *Cdk8*, and *CycC* RNAi knockdowns.

(A, B) Wing discs from larvae with the genotypes *Ms1096-GAL4; UAS-LacZ; RNAi Med12(P{TRiP.HMS01062}attP2* were stained with anti-β-Gal and anti-Med12 antibodies (A), or anti-LexA and anti-Med12 antibodies (B). Med12 RNAi with P{TRiP.HMS01062}attP2 efficiently downregulates the Med12 accumulation according to the Ms1096-GAL4 expression pattern (A), but has no effect on the LexA:Bap170 level (B). (C) Efficient downregulation of Med13 expression via Med13 RNAi with P{TRiP.HMS01305}attP2 activated in the posterior region of the wing disc by en-GAL4. Wing discs were stained with an anti-Med13 antibody. (D–F) The efficiencies of Cdk8 RNAi with Vdrc107187 and CycC RNAi with P{TRiP.HMS01095}attP2 were assessed by monitoring expression of *E(spl)mβ*, a known target of Cdk8 and CycC in third-instar larval wing discs. (D) Normal *E(spl)mβ* expression pattern in a wild-type wing disc. Levels of *E(spl)mβ* expression are reduced in the posterior region of the wing by activating RNAi against Cdk8 (E) or CycC (F). Scale bar, 100 μm.

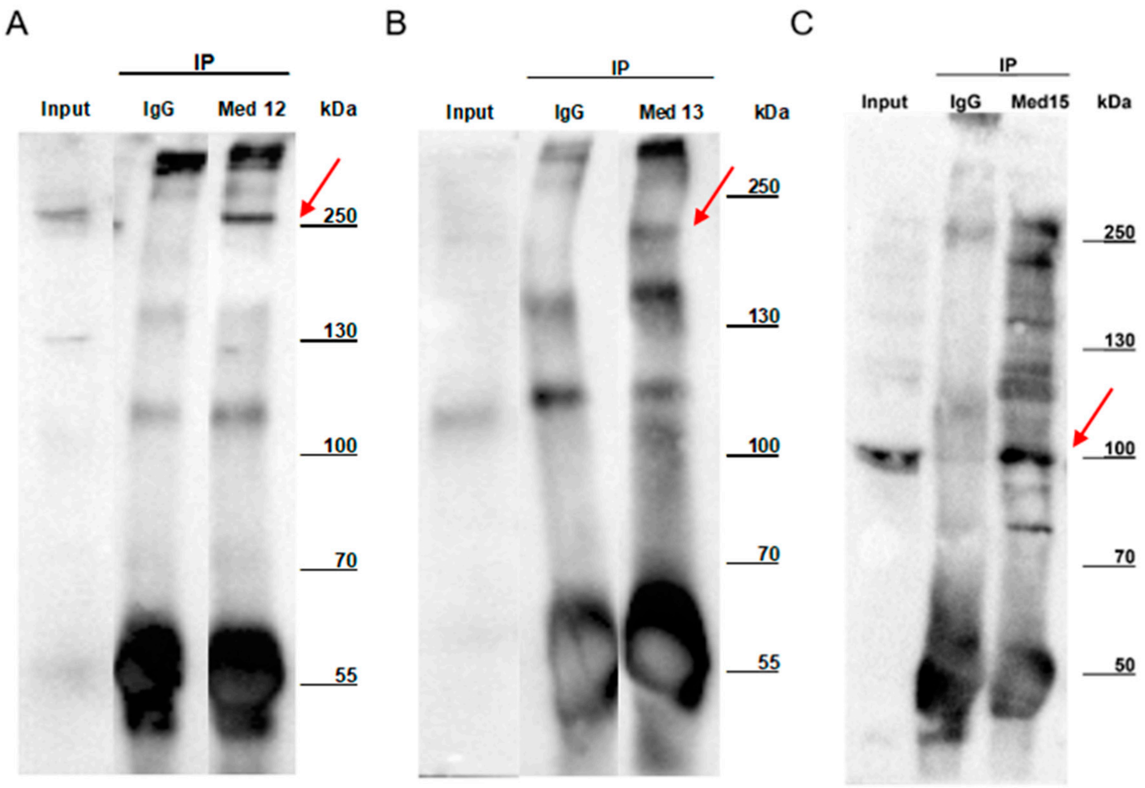

Figure S3. Antibodies against Med12 (A), Med13 (B) and Med15 (C)

Western blot analysis of a total embryonic extract (Input) and immunoprecipitation with anti-Med12 (A) or anti-Med13 (B) antibodies. The corresponding band is indicated with an arrow.

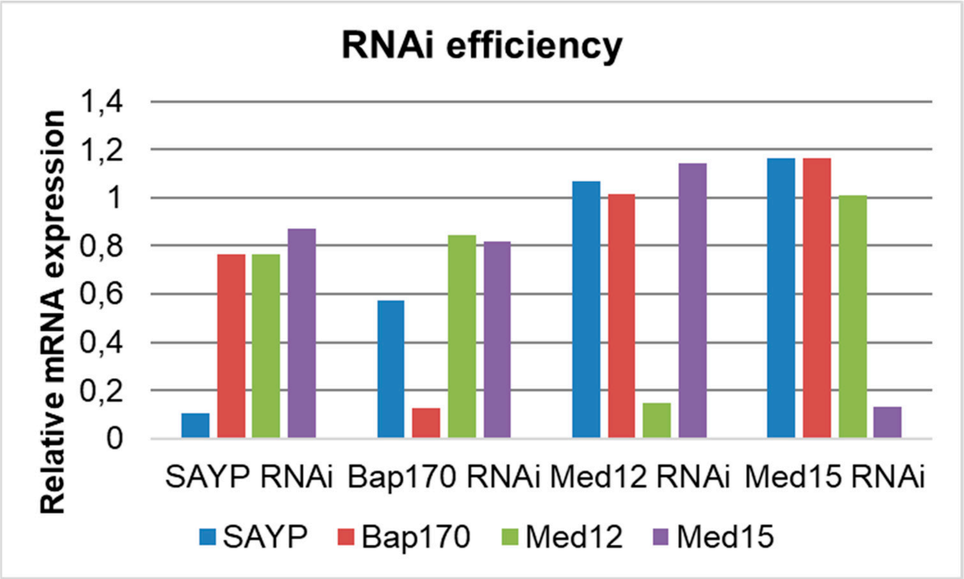

Figure S4. Efficiencies of the *SAYP*, *BAP170*, *Med12*, and *Med15* RNAi knockdowns.

Relative expression of the indicated genes is shown. Expression of the respective gene in untreated cells is taken to be 1.

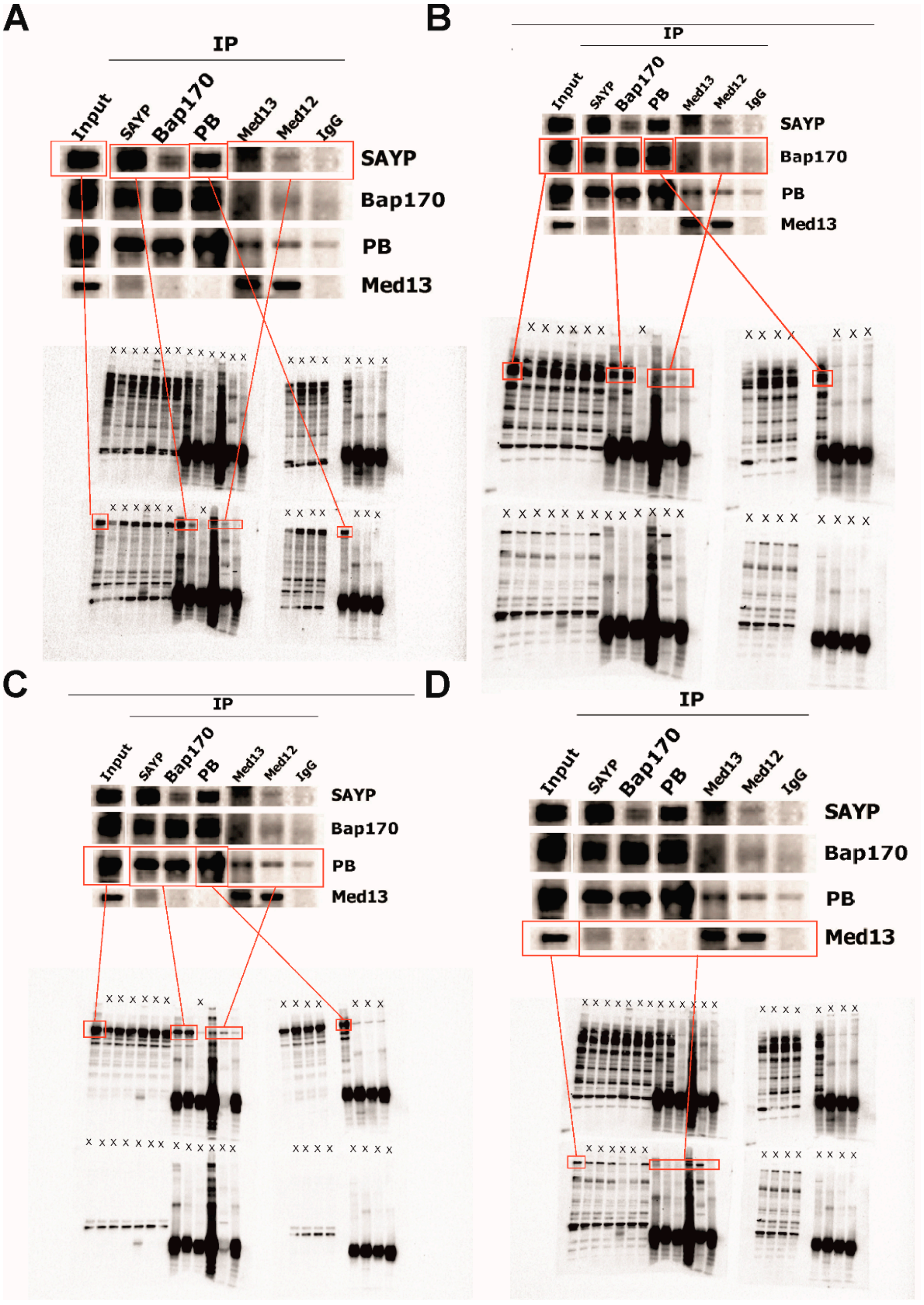

Figure S5. Western blot analysis of co-IP.

Immunoprecipitation from an embryonic nuclear extract was performed with the antibodies indicated at the top; western blot analysis with antibodies against (A) SAYP, (B) Bap170, (C) PB, (D) Med13.

Table S1. Factors checked for effect on SAYP/Bap170-dependent *LacZ* transcription and *taranis* and *Dad* expression.

N.D., not determined; N.E., no effect; ↑, upregulation; ↓, downregulation

	Affected gene	RNAi line	Effect on *LexA:SAYP, LexAop-LacZ*^{Dad} expression	Effect on *LexA:Bap170, LexAop-LacZ*^{Dad} expression	Effect on *Taranis* expression	Effect on *Dad* expression	Phenotype with engrailed-GAL4 (this work)	Phenotype with tubulin-GAL4 (this work)	Other described phenotypes
**MEDIATOR COMPLEX (Head Module)**	*MED6*	*BL33743*	N.D.				prepupal lethality, small imaginal discs	N.D.	wor-GAL4 [79] http://flybase.org/reports/FBal0257745.html
	MED17	*VDRC105264*	N.D.				prepupal lethality, small imaginal discs	N.D.	-
	MED18	*VDRC105264*	partial ↓				wt	N.D.	-
	MED20	*BL34577*	N.E.				pupal lethality, some escapers with severe wing defects	N.D.	-
	MED25	*VDRC108249*	N.E.				wt	N.D.	-
	MED27	*VDRC106703*	N.E.		N.E.	N.E.	prepupal/pupal lethality, some escapers with severe wing defects	N.D.	wor-GAL4 [79] http://flybase.org/reports/FBal0232050.html
	MED28	*VDRC108282*	N.E.	N.E.	N.E.	N.E.	larval lethality	larval lethality	-
**MEDIATOR COMPLEX**	*MED1*	*VDRC13054*	N.E.		N.E.	N.E.	pupal lethality (post eversion)	prepupal lethality	pnr-GAL4 [80] http://flybase.org/reports/FBal0207543.html
	MED4	*VDRC101362*	N.D.		N.E.	N.E.	prepupal/pupal lethality	N.D.	wor-GAL4 [79]

**(Middle Module)**									http://flybase.org/reports/FBal0235721.html
	MED7	*BL34663*	N.D.				prepupal lethality, small imaginal discs	N.D.	-
	MED10	*BL34031*	N.E.				wt	N.D.	wor-GAL4 [79]<br><br>http://flybase.org/reports/FBal0257669.html
	MED21	*VDRC109982*	N.E.				prepupal lethality	N.D.	-
	MED31	*BL34574*	N.E.				wt	N.D.	wor-GAL4 [79]<br><br>http://flybase.org/reports/FBal0257712.html
**MEDIATOR COMPLEX (Tail Module)**	*MED14*	*BL34575*	N.D.				prepupal lethality, small imaginal discs	N.D.	-
	MED15	*VDRC21809*	N.E.			N.E.	extra veins	prepupal/pupal lethality	pnr-GAL4 [80]<br>engrailed-GAL4 [83]<br><br>http://flybase.org/reports/FBal0207547.html
	MED16	*BL34012*	↓	↓	N.E.	N.D.	prepupal lethality	larval lethality	-
**MEDIATOR COMPLEX (Kinase Module)**	*kto (MED12)*	*VDRC23143*	↓	↓	↓	N.E.	prepupal lethality	larval lethality	pnr-GAL4 [80]<br>elav-GAL4 [81]<br>engrailed-GAL4 [83]<br><br>http://flybase.org/reports/FBal0209803.html
		BL34588	↓	↓	↓	N.E.	prepupal lethality	larval lethality	engrailed-GAL4 [83]<br><br>Kto knockdown[16]
	skd (MED13)	*BL34630*	↓	↓	↓	N.E.	prepupal lethality	larval lethality	Skd knockdown [16, 17]
	cdk8	*VDRC107187*	N.E.	N.E.	N.E.	N.E.	extra veins	pupal lethality	-

	cycC	*BL33753*	N.D.	N.E.	N.D.	N.D.	prepupal to pupal lethality, some escapers	larval/pupal lethality	-
		VDRC48835	N.E.	N.E.	N.E.		wt		pnr-GAL4 [80]<br><br>http://flybase.org/reports/FBal0206708.html
**GTF (TFIID)**	*TAF1*	*BL32421*	N.D.				prepupal lethality, small imaginal discs	N.D.	MTD-GAL4 [84]<br><br>http://flybase.org/reports/FBal0248177.html
	TAF4	*VDRC109640*	N.D.		N.D.		prepupal/pupal lethality	N.D.	-
	TAF5	*VDRC45957*	N.D.				prepupal lethality, small imaginal discs	N.D.	pnr-GAL4 [80]<br>elav-GAL4 [81]<br><br>http://flybase.org/reports/FBal0208713.html
		VDRC45955	N.D.				prepupal lethality, small imaginal discs	N.D.	pnr-GAL4 [80]<br>elav-GAL4 [81]<br><br>http://flybase.org/reports/FBal0208713.html
	TBP	*VDRC109756*	N.D.				pupal lethality, some escapers	N.D.	-
**GTF**	*TFIIB*	*VDRC106688*	N.E.				prepupal/pupal lethality	larval lethality	-
**NEGATIVE ELONGATI**	*spt4 (DSIF)*	*VDRC108459*	N.E.	N.E.	N.E.	N.E.	wing defects	pupal lethality	-
	Nelf-A	*VDRC106245*	↓		↓	N.E.	pupal lethality	larval lethality	Nelf-A knockdown [15]

**ON FACTORS**	*Th1 (NELF-D)*	*VDRC100009 Archiviamo perché non agisce sul LexAop.*	N.E.	N.E.	N.E.		prepupal lethality	N.D.	-
	Nelf-E	*VDRC21009*	N.E.	N.D.	N.D.		prepupal/pupal lethality	N.D.	pnr-GAL4 [80]<br>insc-GAL4 [82]<br><br>http://flybase.org/reports/FBal0207733.html
	GAF	*VDRC106433*	↑	N.D.	N.E.		adult wing defects	N.D.	engrailed-GAL4 [83]
**PBAP COMPLEX**	*mor*	*VDRC6969*	↓	↑	↓	N.E.	prepupal lethality	larval/prepupal lethality	pnr-GAL4 [80]<br>Bx-MS1096-GAL4 [78]<br>salm-GAL4 [85]<br>C564-Gal4 and Hml-Gal4 [77]<br><br>http://flybase.org/reports/FBal0210097.html
	Bap170	*VDRC34582*	↓	↓	↓	N.E.	adult wing extra veins	prepupal lethality	pnr-GAL4 [80]<br>salm-GAL4 [85]<br><br>http://flybase.org/reports/FBal0198822.html
	brm	*VDRC37721*	↓	N.E.	N.D.	N.E.	adult wing defects	larval/prepupal lethality	pnr-GAL4 [80]<br>salm-GAL4 [85]<br>insc-GAL4 [82]<br><br>http://flybase.org/reports/FBal0209177.html
	SAYP	*VDRC105946*	↓	N.E.	↓	N.E.	adult wing defects	larval/prepupal lethality	-
	polybromo	*VDRC108618*	N.E.	N.E.	N.E.	N.E.	wt	N.D.	C564-Gal4 and Hml-Gal4 [77]

									http://flybase.org/reports/FBal0231490.html
	snr1	BL32372	N.D.	N.D.	N.D.	N.D.	prepupal lethality, small imaginal discs	N.D.	-

Table S2. List of primers

Primers for dsRNA synthesis

Med12	Forward	CGACTCACTATAGGGAGACGACAACCATTCCAGCAG
	Reverse	CGACTCACTATAGGGAGAGCTGTTGTATCATGCCTTGC
	Forward	CGACTCACTATAGGGAGAAGTCGGAGCGTCTTTG
	Reverse	CGACTCACTATAGGGAGATGGCGCTTGTCAAGTAG
SAYP	Forward	CGACTCACTATAGGGAGATGCCCATCAAAGAATCGG
	Reverse	CGACTCACTATAGGGAGAAAGCGTTCCACTTGGTGG
	Forward	CGACTCACTATAGGGAGAACTGGCGGAGATGCAGAC
	Reverse	CGACTCACTATAGGGAGACCATGTCGTCCGAATTGC
Bap170	Forward	CGACTCACTATAGGGAGACGAGGTAACCGAACGAGG
	Reverse	CGACTCACTATAGGGAGAGCAAGCAGCACATCAAGC
Med15	Forward	CGACTCACTATAGGGAGAAGGTCTGGTAGCCAAGCTC
	Reverse	CGACTCACTATAGGGAGAGGTTCTGCTGAATCTGCTG

Primers for epitope cloning

Med12	Forward	GACACATATGCACTCAACGCTGGTCTC
	Reverse	GATACTCGAGCTTGACTGCACCAAACC
Med13	Forward	GACACATATGCAGACTCCATCGGCAG
	Reverse	GATTCTCGAGTCTCCCTCCTGCTTGG
Med15	Forward	ATGAATTCATGACCGAGGACTGGCAGA
	Reverse	TTAAGCTTAGAGGCAGTTCCGGGGCCA

ChIP

Primers to the *DHR3* promoter have been described in [74].

hsp70 reporter promoter	Forward	TAGCGCTAGCGACGTCGAG
	Reverse	GCTTAGCGACGTGTTCACTTTG
Dad enhancer	Forward	AGGTGAGCGTGTGTTGGTGTG
	Reverse	TCATACATACAGAATGCTTGCGTGC

tara promoter	Forward	CGTTCTCGCTCGCACTCAC
	Reverse	CTCAAGAAGCAGGCAGCAGG
rDNA	Forward	AATTCAGAACTGGCACGGACTTGG
	Reverse	AGAGCACTGGGCAGAAATCACATTG
E75 EcRE	Forward	TATCTGTGTGAGTGGCGCTG
	Reverse	ACTGCTTTCTCGTGGCCTATG
E75B promoter	Forward	GCAGCAGCAGCGACAATC
	Reverse	ATGTTGTTGCTGTCGTTTTGTG
actin 5C promoter	Forward	GGAGAGCATTGCGGCTGATAAG
	Reverse	CTGATGGAGCGGCTTTGTGTC
beta tub56D promoter	Forward	CATGTAGTATGGCCACACTGCG
	Reverse	CTTGGATGTGAACGATTTCCCTC

CDS

E75B	Forward	TGCAATGCAAGAGGTTGCTG
	Reverse	GTCTGCTGCTGCTGCTGTTG
DHR3	Forward	TGCTGAAGACGGGCTCCTTTG
	Reverse	CGAGTCGGATGTGTAGAACGC
SAYP	Forward	TTTAGGCGTGGTCGTTGTG
	Reverse	CCGAAAGGGTTGCTCATC
BAP170	Forward	AATGGATTCCACGCCAGCTC
	Reverse	TTGTTGTTGTGGTGTTTGCGTC
Med12	Forward	TCGCAACAATCCCGACTTTATG
	Reverse	CGCCTGGTTCATGTACTGGTTC
Med15	Forward	GTGAACATGCCACCGAATTTG
	Reverse	TGCTGAGCATCTGCTGGAGAG
